# Regulatory Role of *OsMADS34* in the Determination of Glumes Fate, Grain Yield, and Quality in Rice

**DOI:** 10.3389/fpls.2016.01853

**Published:** 2016-12-15

**Authors:** Deyong Ren, Yuchun Rao, Yujia Leng, Zizhuang Li, Qiankun Xu, Liwen Wu, Zhennan Qiu, Dawei Xue, Dali Zeng, Jiang Hu, Guangheng Zhang, Li Zhu, Zhenyu Gao, Guang Chen, Guojun Dong, Longbiao Guo, Qian Qian

**Affiliations:** ^1^State Key Laboratory of Rice Biology, China National Rice Research InstituteZhejiang, China; ^2^College of Chemistry and Life Sciences, Zhejiang Normal UniversityZhejiang, China; ^3^College of Life and Environmental Sciences, Hangzhou Normal UniversityZhejiang, China

**Keywords:** spikelet, rudimentary glume, sterile lemma, grain size, *OsMADS34*, transcriptional repressor, rice (*Oryza sativa* L.)

## Abstract

Grasses produce seeds on spikelets, a unique type of inflorescence. Despite the importance of grass crops for food, the genetic mechanisms that control spikelet development remain poorly understood. In this study, we used *m34-z*, a new mutant allele of the rice (*Oryza sativa*) E-class gene *OsMADS34*, to examine OsMADS34 function in determining the identities of glumes (rudimentary glume and sterile lemma) and grain size. In the *m34-z* mutant, both the rudimentary glume and sterile lemma were homeotically converted to the lemma-like organ and acquired the lemma identity, suggesting that OsMADS34 plays important roles in the development of glumes. In the *m34-z* mutant, most of the grains from the secondary panicle branches (spb) were decreased in size, compared with grains from wild-type, but no differences were observed in the grains from the primary panicle branches. The amylose content and gel consistency, and a seed-setting rate from the spb were reduced in the *m34-z* mutant. Interesting, transcriptional activity analysis revealed that OsMADS34 protein was a transcription repressor and it may influence grain yield by suppressing the expressions of *BG1*, *GW8*, *GW2*, and *GL7* in the *m34-z* mutant. These findings revealed that *OsMADS34* largely affects grain yield by affecting the size of grains from the secondary branches.

## Introduction

In most flowering plants, flowers consist of sepals, petals, stamens, and pistils, which are arranged in concentric whorls (Ohmori et al., 2009). Genetic and morphological studies of floral homeotic mutants in eudicots, including *Arabidopsis thaliana*, *Antirrhinum majus*, and *Petunia hybrida*, have established the classical ABC model of floral development, and subsequently the ABCDE model, which can also partly explain floral development in grass species (Coen and Meyerowitz, 1991; Jeon et al., 2000; Nagasawa et al., 2003; Ditta et al., 2004; Yamaguchi et al., 2006; Dreni et al., 2007; Li H. et al., 2010). Most of the genes in the ABCDE model encode MIKC^C^-type MADS-domain transcription factors. The temporal and spatial expression patterns of the A, B, C, D, and E-class genes, and their complicated protein interactions, determine the identity and patterning of floral organs (Coen and Meyerowitz, 1991; Pelaz et al., 2000; Theissen and Saedler, 2001; Li H. et al., 2011; Kobayashi et al., 2012; Yun et al., 2013; Hu Y. et al., 2015). In the extended ABCDE model, E-class (*SEPALLATA, SEP*) genes affect the development of floral organs by interacting with and modulating the expression of floral organ-related genes (Jeon et al., 2000; Pelaz et al., 2000; Ferrario et al., 2003; Malcomber and Kellogg, 2004; Liu et al., 2009).

*Arabidopsis thaliana* has four functionally redundant E-class genes (*SEP1*, *SEP2*, *SEP3*, and *SEP4*) that determine floral meristem fate and floral organ identity (Pelaz et al., 2000; Ditta et al., 2004; Li H. et al., 2010). Rice has at least five E-class genes: *LEAFY HULL STERILE1/OsMADS1* (*LHS1*/*OsMADS1*), *OsMADS5*, *OsMADS7*/*OsMADS45*, *OsMADS8*/*OsMADS24*, and *OsMADS34/PAP2* (Jeon et al., 2000; Malcomber and Kellogg, 2005; Kater et al., 2006; Yamaguchi and Hirano, 2006; Gao et al., 2010; Kobayashi et al., 2010). *LHS1*/*OsMADS1* determines the identities of four whorls of floral organs and affects the determinacy of the floral meristem (Jeon et al., 2000; Hu Y. et al., 2015). *OsMADS5* loss-of-function mutants show no obvious phenotypic abnormities (Gao et al., 2010). *OsMADS7* and *OsMADS8* are involved in the regulation of flowering time, floral organ identity, and floral meristem determinacy (Pelucchi et al., 2002; Cui et al., 2010). Silencing of *OsMADS1*, *OsMADS5*, *OsMADS7*, and *OsMADS8* causes homeotic transformation of the palea, lodicule, stamen, and pistil into leaf-like structures (Cui et al., 2010). The *osmads34* mutant displays an abnormal inflorescence with an elongated sterile lemma, suggesting that *OsMADS34* controls spikelet and inflorescence morphology mainly by mainly regulating the identity of the sterile lemma and number of branches of the panicle (Gao et al., 2010; Kobayashi et al., 2010; Lin et al., 2014; Zhang and Yuan, 2014).

As a model monocot plant, rice has unique floral architecture that differs from those of eudicots (Ren et al., 2013). The spikelet is the floral unit in rice and comprises one floret and two pairs of glume-like organs, rudimentary glumes and sterile lemmas (glumes). Generally, the rudimentary glume and sterile lemma are regarded as severely reduced bracts, but their origin has been widely debated (Schmidt and Ambrose, 1998; Ambrose et al., 2000; Hong et al., 2010; Ren et al., 2013). Map-based cloning approaches have helped to elucidate how these organs are specified in the rice spikelet. *LONG STERILE LEMMA*/*ELONGATED EMPTY GLUME* (*G1*/*ELE*) belongs to a plant-specific gene family that encodes an unknown domain protein and is strongly expressed in the sterile lemma primordia. In the *g1*/*ele* mutant, the sterile lemma was homeotically transformed into a lemma-like organ (Yoshida et al., 2009; Hong et al., 2010). *OsMADS34/PAP2* is important for retaining normal sterile lemma identity (Gao et al., 2010; Kobayashi et al., 2010). In the *osmads34/pap2* mutant, normal sterile lemmas were not observed at sites where longer glume-like organs were present. Furthermore, expression of the lemma marker gene *DROOPING LEAF* (*DL*) was detectable in the glume-like organs, indicating that the sterile lemma had acquired the lemma identity (Lin et al., 2014). The *EXTRA GLUME 1* (*EG1*) and *ABERRANT SPIKELET AND PANICLE 1* (*ASP1*) genes determine the identity of the sterile lemma. In the *eg1* and *asp1* mutants, the sterile lemma was elongated and had lemma identity, and the *asp1* mutant also showed enlarged rudimentary glumes and the epidermal structure of rudimentary glumes was similar to that of sterile lemmas, suggesting that the identity was altered (Li et al., 2009; Yoshida et al., 2012). The other class of genes comprises *FRIZZY PANICLE* (*FZP*), *SUPERNUMERARY BRACT* (*SNB*), *OsINDETERMINATE SPIKELET1* (*OsIDS1*), and *MULTI-FLORET SPIKELET1* (*MFS1*), which belong to the APETALA2/ethylene responsive (AP2/ERF) gene family and determine the identities of the rudimentary glumes and/or sterile lemma. Loss of function of *FZP* and *SNB* resulted in extra rudimentary glumes in the mutants, but no sterile lemmas were found in the corresponding position (Komatsu et al., 2003; Lee et al., 2007). A mutation of *OsIDS1* and *MFS1* caused the sterile lemmas to be converted into bract-like organs, which were similar to rudimentary glumes (Lee and An, 2012; Ren et al., 2012, 2013). Although several genes have been successfully isolated and characterized, the identities and origins of the highly derived grass-specific glumes, are still controversial, thus it is necessary to identify more corresponding mutants and isolate these genes involved in regulation of these characteristic organs.

In this study, we discovered a new mutant allele of *OsMADS34* (*m34-z*). The *m34-z* mutant had a unique mutation different from the reported *osmads34* mutants, and *m34-z* caused different phenotypic defects. The *m34-z* mutant had more primary panicle branches (ppb), fewer secondary panicle branches (spb), shorter panicles, and enlarged sterile lemmas, consistent with the phenotypes of the reported *osmads34* mutants. However, the *m34-z* mutant also exhibited elongated rudimentary glumes, small grains, low amylose content (AC) and gel consistency (GC), and a low seed-setting rate from the spb. In addition, *DL* was expressed in the rudimentary glume and sterile lemma in the *m34-z* mutant. These results revealed that both the rudimentary glume and sterile lemma were converted to the lemma-like organs and acquired the lemma identity, indicating that the rudimentary glume, sterile lemma, and lemma may be homologous organs. Our findings also showed that *OsMADS34* is a transcriptional repressor that negatively regulates the expression of genes involved in grain yield and glumes fate. These results indicate that *OsMADS34* plays important roles in the determination of organ identity and affects grain yield and quality.

## Materials and Methods

### Plant Materials

The *m34-z* mutant was a spontaneous mutant whose genetic background was a *japonica* cultivar, Zhonghua (ZH11). The *m34-z* mutant was crossed with the typical *indica* cultivar Nan Jing 6 (NJ6) to construct the mapping population. The obtained F_1_ seeds were sown and transplanted as individual plants to generate the F_2_ plants for gene mapping. ZH11 was used as the wild-type plants for phenotypic analysis. All plants were grown in paddies at the China National Rice Research Institute, Hangzhou and in Lingshui, Hainan Province, China.

### Map-Based Cloning of *OsMADS34*

To create the mapping population, the *m34-z* mutant was crossed with NJ6 and 962 F_2_ plants showing the mutational phenotype were obtained. For initial gene mapping, simple sequence repeat (SSR) markers were developed from public rice databases in the Rice Genomic Research Program and the Gramene websites^1^^,^^2^. Fine mapping was performed using single nucleotide polymorphism (SNP) makers from comparisons of genomic sequences between Nipponbare (a *japonica* cultivar) and 9311 (an *indica* cultivar; Guo et al., 2014). The sequences of the primers used are shown in **Supplementary Table 1**.

**Table 1:** Comparisons of organ size in the wild type and *m34–z* mutant.

	Rg (mm)	SI (mm)	Le (mm)	Pa (mm)
WT-p	0.3–0.5	2.0 ± 0.4	7.3 ± 0.3	6.7 ± 0.4
*m34-z-p*	1.8–7.9	7.9 ± 0.8	7.4 ± 0.2	6.9 ± 0.3
WT-s	0.3–0.5	1.9 ± 0.3	7.2 ± 0.4	6.8 ± 0.2
*m34-z-s*	1.9–7.6	6.2 ± 0.5	6.0 ± 0.3	5.8 ± 0.3


### Microscopy Observations

The paraffin section and scanning electron microscopy (SEM) analyses were carried out as previously described in Ren et al. (2016).

### Characterization of Pollen Sterility

Ten anthers were randomly selected, placed on the slide, and mashed. Pollens were stained with 1% I2-KI solution and photographed with a NIKON ECLIPSE 90i microscope.

### Grain Quality Determination

The GC, gelatinization temperature (GT), and AC were determined as previously described (Su et al., 2011).

### RNA Isolation and Expression Analysis

Total RNA was extracted from roots, culms, leaves, inflorescences with different lengths, developing seeds, and all floral organs of the wild-type and *m34-z* mutant using the RNeasy Plant Mini Kit (Axygen). cDNA was obtained by reverse transcription using the SuperScript III Reverse Transcriptase Kit (Invitrogen) with genomic DNA digestion (Takara) using 2 μg total RNA in a 25 μL reaction volume. qPCR was carried out with the StepOne-Plus System (Applied Biosystems) using the SYBR Green PCR Master Mix kit (Promega). At least three biological replicates were performed for each tissue.

### Complementation Tests

The full coding sequence (CDS) of *OsMADS34* driven by the *Cauliflower mosaic virus* 35S promoter was inserted into the binary vector pCAMBIA1301 to generate the recombinant pCA1301-C plasmid. The pCA1301-C plasmid was transformed into *Agrobacterium tumefaciens* strain LBA4404 and the positive bacterial strains were introduced into the *m34-z* mutants using the *A. tumefaciens*-mediated transformation method (Rao et al., 2015). The primers used are listed in **Supplementary Table 1**.

### GUS Staining

Plant samples (pro*OsMADS34*-GUS) were stained with a solution containing 50 mM NaPO_4_ buffer, 1 mM 5-bromo-4-chloro-3-indolyl-β-D-GlcA, 0.4 mM K_3_Fe(CN)_6_, 0.4 mM K_4_Fe(CN)_6_, and 0.1% (v/v) Triton X-100 (Rao et al., 2015) and incubated at 37°C in the dark for 6–12 h. Chlorophyll was removed from the tissues using an ethanol series. The primer sequences used are listed in **Supplementary Table 1**.

### Subcellular Localization

The coding region of *OsMADS34* without the stop codon was amplified from ZH11 using the primers *OsMADS34*OE-1F and *OsMADS34*OE-1R, which contain SalI sites. Next, the fragment was fused into the 35S-GFP (S65T)-NOS (pCA1301) vector to generate the OsMADS34-GFP recombinant vector using the In-Fusion HD Cloning Kit (Takara). Then, the plasmids of GFP (negative control), AFD1-GFP (positive control), and OsMADS34-GFP were transformed into rice protoplasts and transiently expressed (Ren et al., 2015). After 816 h of incubation at 25–28°C, green fluorescent signals were observed using an OLYMPUS IX71 confocal microscope. The primer sequences used are listed in **Supplementary Table 1**.

### Transcriptional Activity

Transcription activation tests were conducted using the Matchmaker GAL4 Two-Hybrid System 3 (Clontech). The full-length sequence of *OsMADS34* from ZH11 was amplified. For the positive control, the full-length sequence of *OsMADS15* from ZH11 was amplified (Wang et al., 2010). The two target fragments were inserted into the vector pGBKT7 to fuse the GAL4 DNA-binding domain (BD) using the In-Fusion HD Cloning Kit (Takara). All vectors were transformed into yeast strain AH109 and the clones were diluted to an OD600 of 0.5. Then, 1 μl of liquid culture was added to tryptophan-, histidine-, and adenine-negative synthetic dropout medium (Hu J. et al., 2015). The transcriptional activity of OsMADS34 was investigated using the dual luciferase reporter assay system in Arabidopsis protoplasts and the relative luciferase activity was detected as previously described (Wu et al., 2013). The primers used are listed in **Supplementary Table 1**.

## Results

### Phenotypic Defects of the *m34-z* Mutant Spikelet

**FIGURE 1:**
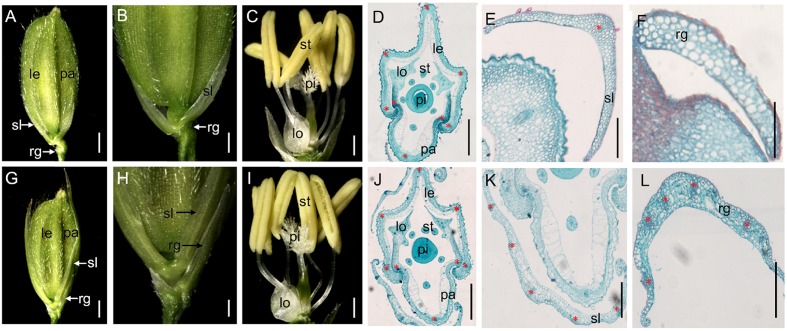
**Phenotypes of spikelets in the wild-type and the *m34-z* mutant.** **(A)** Wild-type spikelet. **(B)** Partial magnification in **(A)**. **(C)** Wild-type floret. **(D)** Histological analysis of the wild-type floret. **(E)** Histological analysis of the sterile lemma in the wild-type spikelet. **(F)** Histological analysis of the rudimentary glume in the wild-type spikelet. **(G)** *m34-z* mutant spikelet. **(H)** Partial magnification in **(G)**. **(I)** *m34-z* mutant floret. **(J)** Histological analysis of the *m34-z* mutant floret. **(K)** Histological analysis of the sterile lemma in the *m34-z* mutant spikelet. **(L)** Histological analysis of the rudimentary glume in the *m34-z* mutant spikelet. rg, rudimentary glume; sl, sterile lemma; le, lemma; pa, palea; lo, lodicule; st, stamen; pi, pistil. Red stars represent vascular bundles. Bars = 1000 μm in **(A,B,G)**, and H; 500 μm in **(C,I)**; and 100 μm in **(D–F,J–L)**.

A normal wild-type rice spikelet consists of two pairs of vestigial glumes (rudimentary glumes and sterile lemmas), which are generated from the spikelet meristem, and one terminal floret comprising a lemma, a palea, two lodicules, six stamens, and a pistil (**Figures 1A–D**).

In the vegetative phase, we observed no obvious defects in the *m34-z* mutant. However, in the reproductive phase, we observed significant abnormalities in the *m34-z* mutant after heading. First, 56% of the *m34-z* mutant spikelets showed longer rudimentary glumes which were different from the reported phenotypes of the other *osmads34* mutants. The wild-type rudimentary glumes averaged about 0.5 mm in length, but the sizes of*m34-z* mutant rudimentary glumes were various from about 1.8– 7.9 mm in length (**Figures 1A,B,G,H,L**; **Table 1**). In the *m34-z* mutant spikelets with serious defects, the rudimentary glumes resembled sterile lemmas or lemmas and were similar in size to the wild-type lemmas (**Table 1**). Second, 92% of the *m34-z* mutant spikelets developed larger sterile lemmas that were indistinguishable from the lemmas of the wild-type or the *m34-z* mutant (**Figures 1G,E,K**; **Table 1**).

**FIGURE 2:**
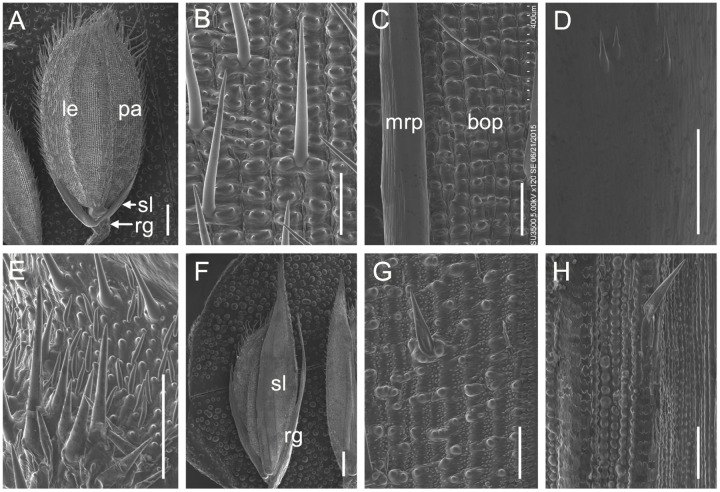
**scanning electron microscopy (SEM) analysis of glumes in the wild-type and the *m34-z* mutant at heading stage.** **(A–E)** Wild-type spikelet. **(B)** Epidermal surface of the wild-type lemma. **(C)** Epidermal surface of the wild-type palea. **(D)** Epidermal surface of the wild-type sterile lemma. **(E)** Epidermal surface of the wild-type rudimentary glume. **(F–H)** *m34-z* mutant spikelet. **(G)** Epidermal surface of the *m34-z* mutant sterile lemma. **(H)** Epidermal surface of the *m34-z* mutant rudimentary glume. rg, rudimentary glume; sl, sterile lemma; le, lemma; pa, palea; bop, body of palea; mrp, marginal region of palea. Bars = 1000 μm in **(A,F)** and 100 μm in **(B–E**,**G–H)**.

We also investigated the floral organs of four whorls and these organs appeared normal in the *m34-z* mutant (**Figures 1A,C,D,G,I,J**). We performed paraffin section and SEM analysis on the structures of the spikelets in the *m34-z* mutant and wild-type plants. The wild-type lemma and palea [which comprises the marginal regions of the palea (mrp) and the body of the palea (bop)] developed five and three vascular bundles, respectively (**Figures 1D** and **2C**). The wild-type lemma and bop had four cell layers, including non-silicified cells, spongy parenchymatous cells, fibrous sclerenchyma, and silicified cells (**Supplementary Figure 1**), and the mrp exhibited a smooth epidermis but lacked silicified epicuticular cells (**Figure 2C**). In the wild-type spikelets, the sterile lemma developed one vascular bundle, showed a smooth epidermis and regularly arranged cells (**Figures 1E** and **2D**), and rare trichomes were observed on the epidermis (**Figure 2D**). The epidermis of the rudimentary glumes of the wild-type showed irregularly arranged cells with numerous, small protrusions, and trichomes (**Figure 2E**). No obvious vascular bundles were observed in the rudimentary glumes of the wild-type (**Figure 1F**). In contrast, the sterile lemmas of the *m34-z* mutant were elongated and developed five vascular bundles (**Figures 1G,K** and **2F**), and they exhibited a similar histological structure to that of the wild-type lemma or bop including protrusions and trichomes (**Figures 2B,C,G**). In the *m34-z* mutant spikelets, the rudimentary glumes were elongated, had five vascular bundles, and contained large protrusions and trichomes (**Figures 1L** and **2F,H**), similar to that of the wild-type lemma or bop.

**FIGURE 3:**
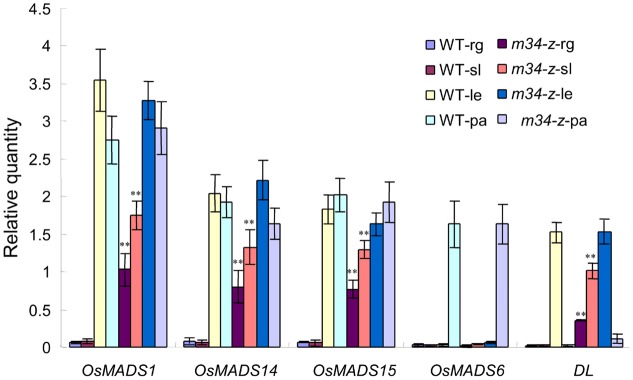
**Relative expression levels of genes involved in floral organ development in the wild-type and the *m34-z* mutant spikelet.** rg, rudimentary glume; sl, sterile lemma; le, lemma; pa, palea; WT, wild-type. Error bars indicate SD. ^∗∗^Significant difference at *P* < 0.01 compared with the wild-type by Student’s test.

Next, we investigated the expressions of the lemma marker gene *DL*, the hull (lemma and palea) marker genes *OsMADS1*, *OsMADS14*, and *OsMADS15*, and the palea marker gene *OsMADS6* in the *m34-z* mutant sterile lemmas and rudimentary glumes. In the wild-type, the transcripts of *OsMADS1*, *OsMADS14*, and *OsMADS15* were found in the lemma and palea. *DL* and *OsMADS6* was mainly expressed in the lemma and palea, respectively. However, no signals of all these genes were observed in the sterile lemma and rudimentary glume. Whereas, the expression of *OsMADS1*, *OsMADS14*, *OsMADS15*, and *DL* increased in the *m34-z* mutant compared with wild-type, but transcripts of *OsMADS6* were not detected in the sterile lemma and rudimentary glume of *m34-z* mutant (**Figure 3**). These results indicated that the rudimentary glumes and sterile lemmas of the *m34-z* mutant were converted to lemma-like organs and partly acquired the identity of the lemma but not the identity of the palea.

### Abnormal Early Spikelet Development in the *m34-z* Mutant

**FIGURE 4:**
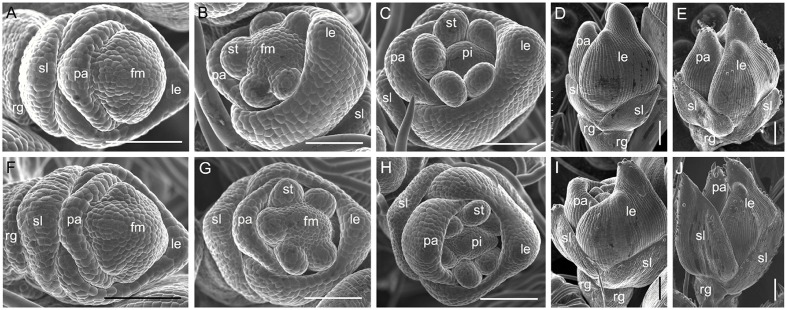
**Spikelets at early developmental stages in the wild-type and the *m34-z* mutant.** **(A–E)** wild-type spikelet. **(A)** Sp4, **(B)** Sp5-6, **(C)** Sp7, **(D)** Sp8, **(E)** After Sp8, **(F–I)** *m34-z* mutant spikelet, **(F)** Sp4, **(G)** Sp5-6, **(H)** Sp7, **(I)** Sp8, **(J)** After Sp8. fm, floral meristem; rg, rudimentary glume; sl, sterile lemma; le, lemma; pa, palea; st, stamen; pi, pistil. Bars = 100 μm.

We analyzed young spikelets at different developmental stages to further investigate spikelet development in the *m34-z* mutant. During the spikelet 4 stage (Sp4) of development in the wild-type, the sterile lemma and rudimentary glume primordia were formed and the palea and lemma primordia were developing (**Figure 4A**). At the Sp5 and Sp6 stages, six stamen primordia were found in the wild-type spikelet and the stamen primordium on the lemma side was delayed (**Figure 4B**). At these stages, the growth of the rudimentary glume ceased and the sterile lemma continued growing. During the Sp7 and Sp8 stages in the wild-type spikelet, the pistil primordium was formed and the sterile lemma further differentiated, becoming much longer than the rudimentary glume (**Figures 4C,D**). In contrast, the sterile lemma of the *m34-z* mutant spikelets showed obvious morphological differences from that of the wild-type. At Sp4 through Sp8, the *m34-z* mutant spikelets displayed larger sterile lemmas than those of the wild-type (**Figures 4F–I**). After the Sp8 stage, we found a few small protrusions on the epidermis of the wild-type and *m34-z* spikelets. And the sterile lemma of the *m34-z* mutant was dramatically larger than that of the wild-type and the size of the sterile lemma of the *m34-z* mutant was comparable to the lemma of the wild-type or *m34-z* mutant (**Figures 4E,J**). However, we found no obvious differences between the rudimentary glumes of the wild-type and the *m34-z* mutant at from Sp4 through Sp8 (**Figures 4A–J**). Also, we did not observe significant defects in floral organs in the *m34-z* mutant, including the lemma, palea, lodicule, stamen, and pistil (**Figures 4A–C,F–H**). These results revealed that *OsMADS34* affects the initiation and enlargement of the sterile lemma but does not influence the development of floral organs. Because longer rudimentary glumes were observed in the wild-type at the heading stage, we speculated that *OsMADS34* may be involved in regulation of the rudimentary glume at later stages.

### The *m34-z* Mutant Has Reduced Grain Yield and Quality

We compared agronomic traits between the wild-type and the *m34-z* mutant at grain maturation. The *m34-z* mutant displayed shorter panicles, more ppb, and fewer spb compared to the wild-type (**Supplementary Figures 2B,C**). A lower seed-setting rate was also found in the *m34-z* mutant. The total seed-setting rate was only 73% in the *m34-z* mutant, whereas it reached up to 86% in the wild-type (**Supplementary Figure 2E**). The seed-setting rate of the ppb in the *m34-z* mutant was comparable with that in the wild-type. However, the seed-setting rate of the spb in the *m34-z* mutant was 65%, whereas the seed-setting rate of the spb in the wild-type was 84% (**Supplementary Figure 2E**). This finding indicated that the low seed-setting rate in the *m34-z* mutant could be attributed to the lower seed-setting rate of the spb.

**FIGURE 5:**
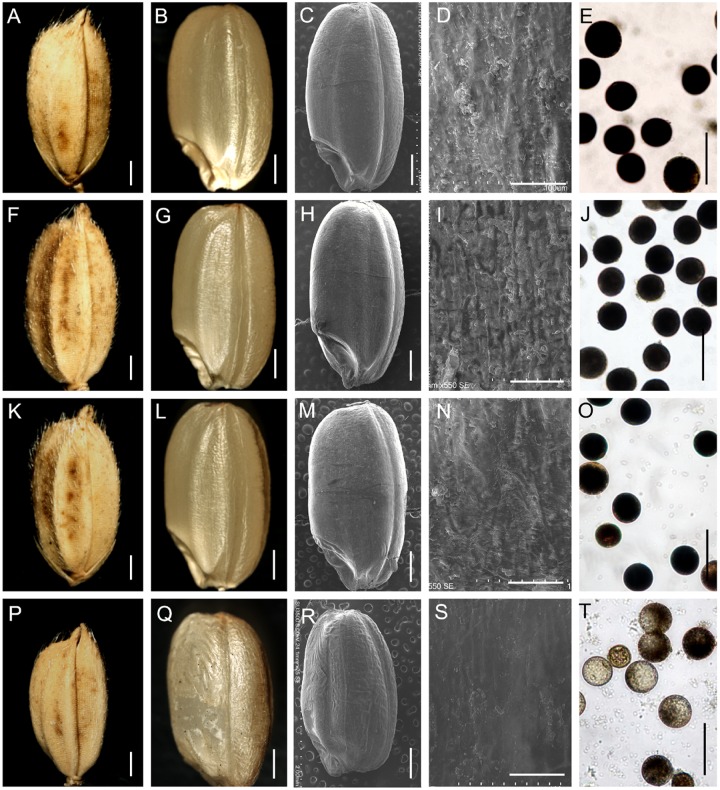
**Phenotypic observations of grain and pollen viability in the wild-type and the *m34-z* mutant.** **(A–E)** Phenotypes of the grain and pollen viability from the primary panicle branches (ppb) in the wild-type. **(F–J)** Phenotypes of the grain and pollen viability from the ppb in the *m34-z* mutant. **(K–O)** Phenotypes of the grain and pollen viability from the secondary panicle branches (spb) in the wild-type. **(P–T)** Phenotypes of the grain and pollen viability from the spb in the *m34-z* mutant. **(A,F,K,P)** grain size. **(B,G,L,Q)** size of brown rice. **(C–D,H–I,M–N,R–S)** epidermal surface of brown rice. E, J, O, and T, pollen viability. Bars = 1000 μm in **(A–C,F–H,K–M)**, and P-R; 50 μm in **(D,I,N,S)**; 100 μm in **(E,J,O,T)**.

Next, an I_2_-KI test showed that the viability of the pollen from the stamens of the ppb of the *m34-z* mutant spikelets was not altered, but the viability of the pollen from the spb was decreased (**Figures 5E,J,O,T**; **Supplementary Figure 2D**), which was consistent with the low seed-setting rate of the spb in the *m34-z* mutant. The grains from the spb were smaller in the *m34-z* mutant compared to the wild-type, but the grains from the ppb in the *m34-z* mutant were normal in size (**Figures 5A–C,F–H,K–M,P–R**; **Supplementary Figure 2A**). The average lengths of the wild-type grains and brown rice (grains from which the hull was removed) were 7.0 and 5.6 mm, respectively, while the average lengths of the *m34-z* mutant grains and brown rice were 6.0 and 4.5 mm, respectively (**Figures 5K,L,P,Q**; **Supplementary Figures 2A** and **3**). The average widths of the *m34-z* mutant grains and brown rice were similar to those of the wild-type (**Supplementary Figure 3**). Moreover, the 1,000-grain weight and weight of 1,000 brown rice from the spb of the *m34-z* mutant were markedly decreased compared to the wild-type, but the 1,000-grain weight and weight of 1,000 brown rice from the ppb were not changed (**Supplementary Figure 3**).

**FIGURE 6:**
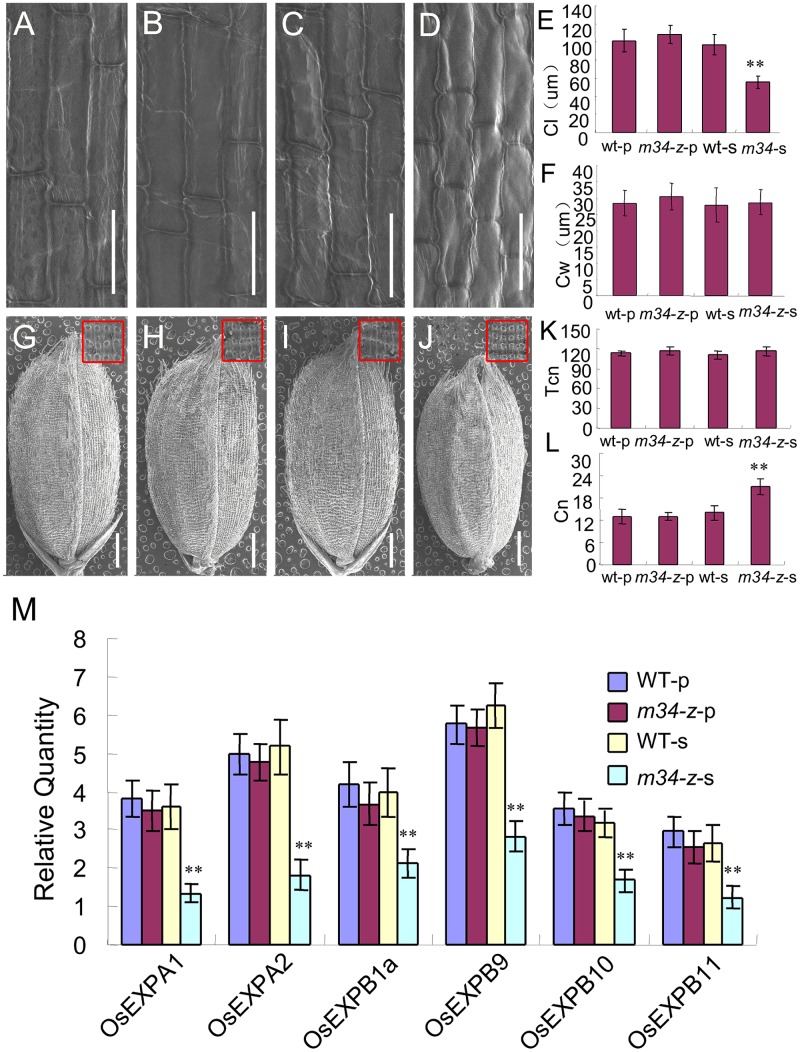
***OsMADS34* influences cell expansion.** **(A)** Inner epidermal cells of hulls from the ppb in the wild-type. **(B)** Inner epidermal cells of hulls from the ppb in the *m34-z* mutant. **(C)** Inner epidermal cells of hulls from the spb in the wild-type. **(D)** Inner epidermal cells of hulls from the spb in the *m34-z* mutant. **(E)** Cell length. **(F)** Cell width. **(G)** Grain from the ppb in the wild-type. **(H)** Grain from the ppb in the *m34-z* mutant. **(I)** Grain from the spb in the wild-type. **(J)** Grain from the spb in the *m34-z* mutant. **(K)** Total cell number along the longitudinal axis of the lemma. **(L)** Total cell number per millimeter along the longitudinal axis of the lemma. **(M)** Expression analysis of cell expansion-related genes in young panicles. WT-p, grains from the ppb in the wild-type; WT-s, grains from the spb in the wild-type; *m34-z*-p, grains from the ppb in the *m34-z* mutant; *m34-z*-s, grains from the spb in the *m34-z* mutant. The red boxes indicate the middle part of the lemma in **(G,H,I,J)**. Bars = 50 μm in **(A–D)**. Error bars indicate SD. ^∗∗^Significant difference at *P* < 0.01 compared with the wild-type by Student’s test.

To investigate the cellular basis for the smaller grains from the spb of the *m34-z* mutant, we measured the cell size from the middle part of the lemmas by SEM. The average cell lengths and widths of the lemmas from the ppb of the *m34-z* mutant were similar to those of the wild-type (**Figures 6A–F**). However, the average cell lengths were reduced in the lemmas from the spb of the *m34-z* mutant compared to the wild-type, but the average cell widths were unchanged (**Figures 6A–F**). We also investigated cell number in the outer epidermis of the lemmas and found no differences in total cell number along the longitudinal axis of the lemmas from the ppb and spb of the wild-type and *m34-z* mutant (**Figures 6G–K**). However, the number of cells per millimeter along the longitudinal axis of the lemmas from the spb of the *m34-z* mutant was significantly higher than that of the wild-type (**Figures 6G–J,L**).

Next, we examined the transcript levels of 35 genes involved in the regulation of cell cycle and cell expansion in rice. Among them, the expression of six cell expansion-related genes was decreased in the spb of the *m34-z* mutant and no differences were detected in the ppb of the *m34-z* mutant (**Figure 6M**). These results suggest that *OsMADS34* mainly controls grain size on the spb by regulating cell expansion but not cell number. *GN1*, *DEP1*, *DEP2*, *DEP3*, *APO1*, and *NAL1* are closely associated with panicle architecture in rice (Ashikari et al., 2005; Ikeda et al., 2007; Huang et al., 2009; Li F. et al., 2010; Qiao et al., 2011; Fujita et al., 2013). We investigated the expression levels of these genes in young panicles of the *m34-z* mutant and the wild-type. *DEP1*, *DEP2*, and *DEP3* were up-regulated compared to the control, and the expression levels of *GN1a*, *APO1*, and *NAL1* were not changed in the *m34-z* mutant (**Supplementary Figure 4**). We also detected the expression levels of genes related to grain size in the wild-type and the *m34-z* mutant (Song et al., 2007; Wang et al., 2008, 2012; Mao et al., 2010; Li Y. et al., 2011; Chen et al., 2013; Hu J. et al., 2015; Liu L. et al., 2015; Liu S. et al., 2015; Wang S. et al., 2015; Wang Y. et al., 2015). Compared with the wild-type, *BG1*, *GW2*, *GW8*, and *GL7/GW7* were up-regulated in the *m34-z* mutant (**Supplementary Figure 4**), indicating that *OsMADS34* negatively regulates the expression of these genes involved in grain size. These findings supported the phenotypic observations and the hypothesis that *OsMADS34* may influence panicle architecture and grain size by negatively regulating the expression.

Next, we studied the epidermis of brown rice. Compared with the wild-type, the epidermis of brown rice from the spb of the *m34-z* mutant was easily wrinkled (**Figures 5C,D,H,I,M,N,R,S**), but the epidermis of brown rice from the spb in the *m34-z* mutant showed rare protrusion-like structures (**Figures 5M,N,R,S**). No differences in the epidermis of brown rice from the ppb were detected between the wild-type and the *m34-z* mutant (**Figures 5C,D,H,I**). Next, we examined the GC, GT, and amylase content (AC) of the grains from the wild-type and the *m34-z* mutant. In the grains from the spb of the *m34-z* mutant, AC was reduced by 17% compared to that in the wild-type, the GC was slightly reduced compared to that in the wild-type, but GT was not altered (**Supplementary Table 2**). No significant differences were found in the GC, GT, and AC of grains from the ppb (**Supplementary Table 2**). Thus, the mutation in *OsMADS34* in the *m34-z* mutant results in reduced grain quality from the secondary branches.

### Identification of OsMADS34 As Responsible for the m34-z Phenotype

**FIGURE 7:**
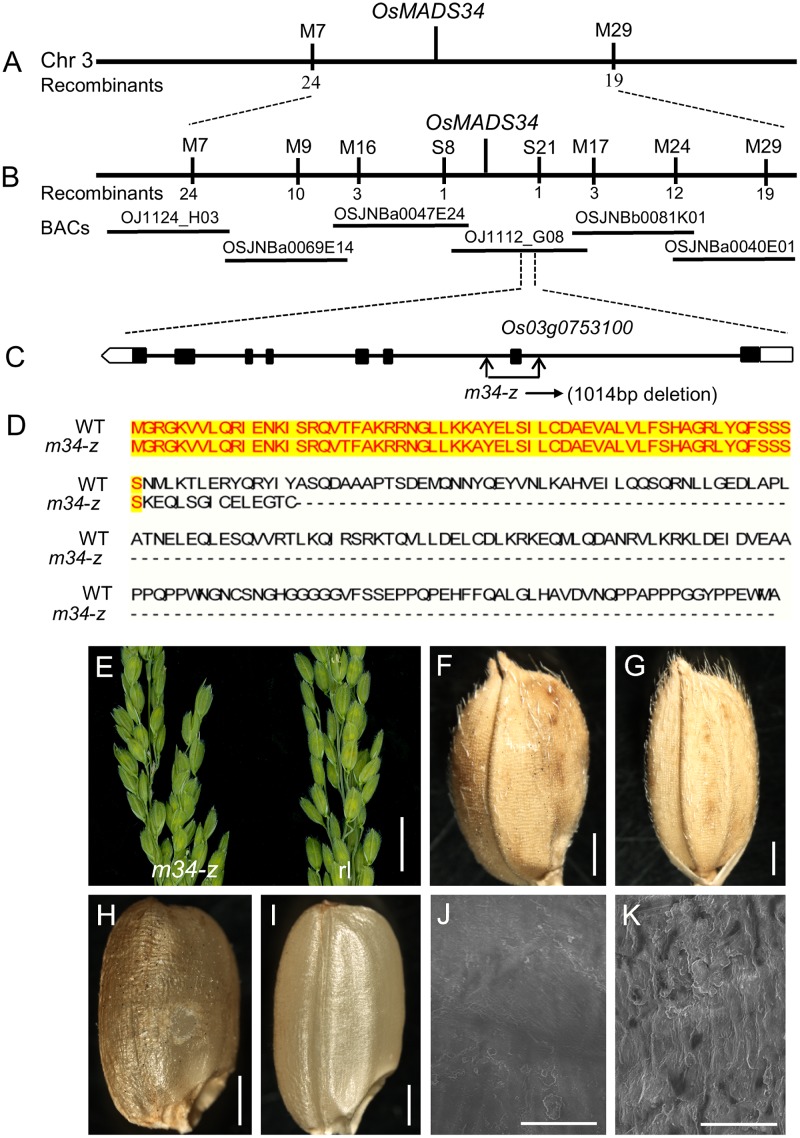
**Identification of OsMADS34 gene as the causal gene for the the *m34-z* mutant.** **(A–C)** Map position of the *OsMADS34* locus. The relative position of the BAC clone is shown. Genomic structure of *OsMADS34*. The mutated site of the *m34-z* mutant is shown. **(D)** *OsMADS34* encodes a 239 amino acid protein, while the mutation of *OsMADS34* in the *m34-z* mutant encodes a 76 amino acid protein due to a coding frame-shift. **(E–K)** Complementation test. All mutant phenotypes (deformed spikelets, reduced grains, and abnormal epidermal surface) were rescued in the transgenic plants. **(E)** Deformed spikelets were recovered in the rescued lines. **(F,H)** Grains from the spb in the *m34-z* mutant. **(G,I)** Reduced grains from the spb in the *m34-z* mutant were rescued in the transgenic lines. **(J)** Epidermal surface of brown rice from the spb in the *m34-z* mutant. **(K)** Epidermal surface of brown rice from the spb in the *m34-z* mutant was similar with that of the wild-type in the transgenic lines. Bars = 1 cm in **(E)**; 1000 μm in **(F–I)**; 50 μm in **(J–K)**.

To clone the locus responsible for the *m34-z* mutant phenotype, we performed a cross between the *m34-z* mutant and *indica* cultivar NJ6. Among the F_2_ plants, 2,736 displayed the normal phenotype and 962 plants displayed the *m34-z* mutant phenotype, a segregation ratio of approximately 3:1, indicating that the mutant phenotype was controlled by a single recessive nuclear gene. We used the 962 recessive mutant plants for the mapping population. Among the 215 SSR markers used in this study, which were evenly distributed throughout the 12 chromosomes, 130 were polymorphic between the two parental lines, and the mutated locus was preliminarily mapped on chromosome 3 between the markers M7 and M29 (**Figure 7A**). For fine mapping of *OsMADS34*, 36 SNP markers were used and six markers displayed polymorphisms (**Figure 7B**). The location of *OsMADS34* was narrowed to a 78 kb distance between the two markers S8 and S21 (**Figure 7B**). We identified a 1,014 bp deletion in *Os03g0753100* (*OsMADS34*) by sequencing analysis, which triggered a premature translation stop (**Figures 7C,D**). To determine whether *Os03g0753100* was causally linked to the *m34-z* mutant phenotypes, the coding region of *OsMADS34* driven by the *Cauliflower mosaic virus* 35S promoter was transformed into calli derived from *m34-z* mutant seeds. Eighteen transgenic lines were obtained and all phenotypic defects were rescued in each line, including the elongated rudimentary glumes and sterile lemmas, and the reduced seed set from the spb (**Figures 7E–K**; **Supplementary Figures 5A,B**).

### Expression Patterns, Subcellular Localization, and Transcriptional Activity Analysis

We investigated the expression of *OsMADS34* in the wild-type and *m34-z* mutant to determine its expression patterns. *OsMADS34* transcripts appeared in all examined tissues and organs including roots, culms, leaves, panicles of different lengths, developing seeds, rudimentary glumes, sterile lemmas, lemmas, paleae, lodicules, stamens, and pistils in the wild-type (**Figure 9A**). And the expression level was higher in young panicles, developing seeds, rudimentary glumes, sterile lemmas, and lemmas. However, much lower expressions were found in tested vegetative and reproductive tissues in the *m34-z* mutant.

To investigate the tissue specificity of *OsMADS34* expression, we fused the *OsMADS34* promoter region with the β-glucuronidase (GUS) reporter gene and transformed the construct into ZH11. GUS signals from pro*OsMADS34-GUS* were detected in the root, culm, leaf, and panicle (**Figures 9B–E**). However, the signals were weak in the root, culm, and leaf, and strong in the panicle (**Figures 9B–E**). In previous studies, *in situ* hybridization revealed that the *OsMADS34* mRNA mainly accumulated in the various stages of inflorescence development (Kobayashi et al., 2010). Subsequently, strong *OsMADS34* signals were detected in the spikelet and floral primordia, such as rudimentary glumes, sterile lemmas, and four whorls of floral organs (Kobayashi et al., 2010). These results were consistent with our qPCR analysis and GUS staining, and supported the phenotypic observations.

**FIGURE 8:**
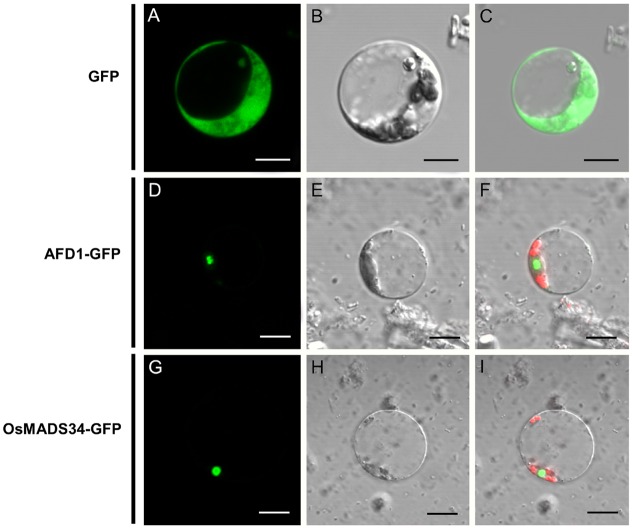
**Subcellular localization of the OsMADS34 protein.** **(A–C)** GFP fusion protein. **(A)** differential interference contrast (DIC) image; **(B)** bright-field image; **(C)** merged image of GFP fusion protein. **(D–F)** AFD1-GFP. **(D)** DIC image; **(E)** bright-field image; **(F)** merged image of AFD1-GFP fusion protein. **(G–I)** OsMADS34-GFP. **(G)** DIC image; **(H)** bright-field image; **(I)** merged image of OsMADS34-GFP fusion protein. Bars = 5 μm in **(B–G)**.

We also examined the subcellular localization of the OsMADS34 protein. The fusion proteins, including OsMADS34-GFP, AFD1-GFP (positive nuclear protein control, Ren et al., 2015), and single GFP, were transiently expressed in rice protoplasts. In the cells that were transformed with single GFP, the green fluorescence was visible in whole cells and the signals surrounded the cell nucleus, cytoplasm, and cell membrane (**Figures 8A–C**). In the cells that were transformed with OsMADS34-GFP and AFD1-GFP, the green fluorescence was exclusively found in the nuclei (**Figures 8D–I**), suggesting that OsMADS34 is predominantly a nuclear protein.

**FIGURE 9:**
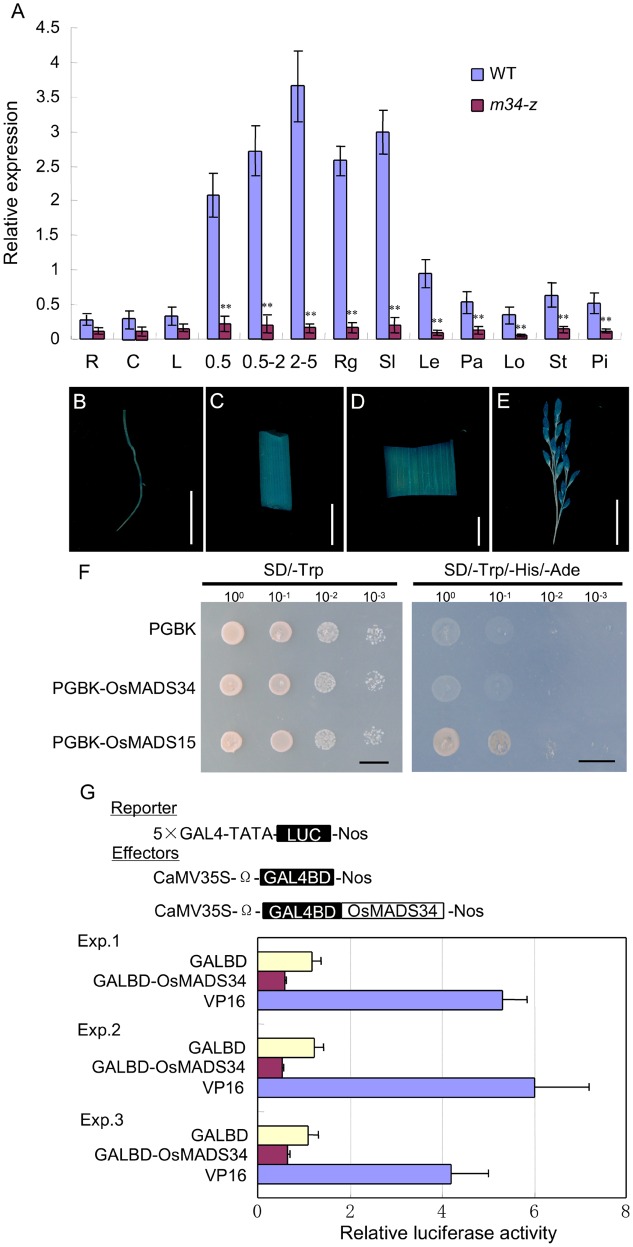
**Expression patterns and transcriptional activity analysis.** **(A)** *OsMADS34* expression in different tissues detected by qPCR. **(B)** GUS staining of root. **(C)** GUS staining of culm. **(D)** GUS staining of leaf. **(E)** GUS staining of spikelet. **(F,G)** Transcriptional activity test. R, root; C, culm; L, leaf; Rg, rudimentary glume; Sl, sterile lemma; Le, lemma; Pa, palea; Lo, lodicule; st, stamen; pi, pistil; 0.5 cm, young panicles (≤0.5 cm); 0.5–2 cm, young panicles (0.5–2 cm); 2–5 cm, young panicles (2–5 cm); 1D, 1 day after pollination; 4D, 4 days after pollination; 7D, 7 days after pollination; 10D, 10 days after pollination. Bars = 1 cm in **(B–G)**. Error bars indicate SD. ^∗∗^Significant difference at *P* < 0.01 compared with the wild-type by Student’s test.

Next, we assayed the transcriptional activation activity of OsMADS34 protein. The coding regions for OsMADS34 and the known transcriptional activator OsMADS15 were fused to the DNA-BD of yeast GAL4. The empty pGBKT7 vector and BD-OsMADS15 were regarded as the negative control and positive control, respectively (Wang et al., 2010). BD-OsMADS15 enabled transformed yeast cells to grow on histidine-deficient medium, but yeast cells harboring BD-OsMADS34 or the empty pGBKT7 vector could not survive on the histidine-deficient medium (**Figure 9F**), suggesting that OsMADS34 does not have transcription activation activity.

We further analyzed the transcriptional activity using a dual luciferase reporter assay in transient assays in Arabidopsis protoplasts. We used a luciferase (LUC) reporter gene that contains five copies of binding sites for GAL4. As in the yeast assays, the coding region of OsMADS34 was fused in-frame to the GAL4 DNA-BD, but the construct was driven by the constitutive 35S promoter of *Cauliflower mosaic virus* (BD-OsMADS34; **Figure 9G**). The GAL4-BD was used as the negative control and VP16 was used as a transcriptional activator (Wu et al., 2013). Three effectors, GAL4-BD, BD-OsMADS34, and VP16, were expressed transiently in Arabidopsis protoplasts. OsMADS34 showed less luciferase activity compared with GAL4-BD and VP16 (**Figure 9G**). These results demonstrated that OsMADS34 functions as a transcriptional repressor.

### *OsMADS34* Affects the Expression of Genes Related to Glume Development

**FIGURE 10:**
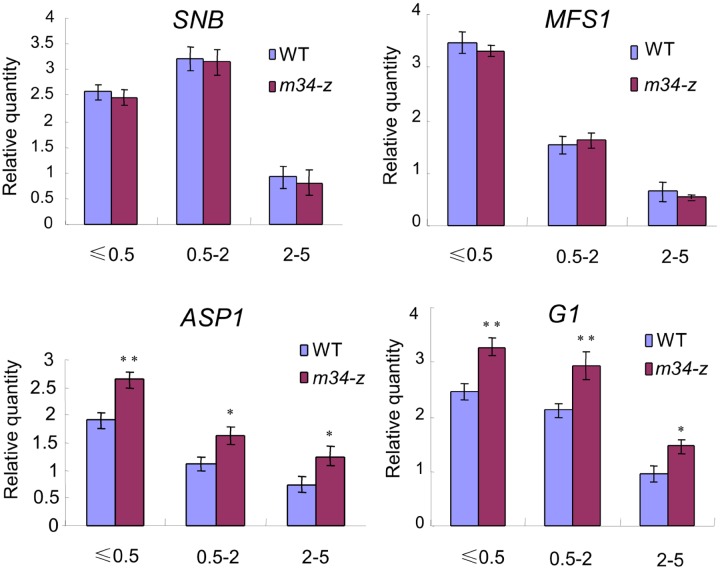
**Expression of genes related to glume development at different stages.** 0.5 cm, young panicles (≤0.5 cm); 0.5–2 cm, young panicles (0.5–2 cm); 2–5 cm, young panicles (2–5 cm). Error bars indicate SD. **Significant difference at *P* < 0.01 compared with the wild-type by Student’s test; *Significant difference at *P* < 0.05 compared with the wild-type by Student’s test.

Because the *m34-z* mutant exhibited defects in glume development, we investigated if OsMADS34 regulates the expression of genes known to be associated with glume (rudimentary glume and sterile lemma) specification and spikelet development in rice. We examined the transcript levels of *G1*, *ASP1*, *MFS1*, and *SNB* in young panicles less than 0.5 cm, between 0.5 and 2 cm, and between 2 and 5 cm in length from the wild-type and *m34-z* mutant (Lee et al., 2007; Yoshida et al., 2009, 2012; Hong et al., 2010; Lee and An, 2012; Ren et al., 2013). In the wild-type, abundant levels of *G1* and *ASP1* transcripts were found in the panicles less than 2 cm long, and their transcript levels decreased markedly in the panicles with a length of 2–5 cm (**Figure 10**). Higher levels of expression of *G1* and *ASP1* were detected in the *m34-z* mutant in all tested panicles compared to the wild-type (**Figure 10**). However, the expression levels of *SNB* and *MFS1* were not altered in the *m34-z* mutant (**Figure 10**). These results revealed that the abnormal glumes in the *m34-z* mutant may be related to altered levels of transcription of glume and spikelet development-related genes, and *OsMADS34* may determine glumes fate by negatively modulating the expressions of *G1* and *ASP1*.

## Discussion

### OsMADS34 Affects Grain Size and Quality in Rice

Grain size and weight are important agronomic traits for yield and quality in cereal crops (Zuo and Li, 2013). Although several genes that control rice grain shape (*GW2*, *GW8*, GS5, *GL7/GW7*, *BG1*, and *DSG1*) have been cloned (Song et al., 2007; Li Y. et al., 2011; Wang et al., 2012; Liu L. et al., 2015; Wang S. et al., 2015; Wang Y. et al., 2015), the complicated genetic mechanisms and regulatory networks by which grain shape is determined remain unknown.

The *m34-z* mutant produced small, short grains from the spb, but normal grains from the ppb. Meantime, qPCR analysis showed that the expressions of *BG1*, *GW8*, *GW2*, and *GL7* were dramatically increased in the *m34-z* spb and no obvious differences were observed in *m34-z* ppb compared with the wild-type, which supported abnormal phenotypes in the *m34-z* mutant. These results suggest that *OsMADS34* may be a regulatory factor that modulates grain size in the spb in rice. Cell number and cell size determine organ size via cell proliferation and cell expansion, respectively (Jiang et al., 2012). In the *m34-z* mutant, the hulls from the spb had smaller cells. No difference in cell number was found between the wild-type and the *m34-z* mutant. qPCR analyses showed that *OsMADS34* regulated the expression of cell expansion-related genes, in agreement with the phenotypic observations. These findings indicated that *OsMADS34* affects grain size by regulating cell expansion. Also, the *m34-z* mutant produced shorter panicles, more ppb, and fewer spb which mimicked the phenotype of *osmads34-1* mutant (Gao et al., 2010), implying that *OsMADS34* influences rice panicle architecture. Interesting, transcriptional activity analysis revealed that OsMADS34 protein was a transcription repressor and the expressions of several genes involved in the regulation of grain size and panicle shape were obviously up-regulated in the *m34-z* mutant. These findings suggested that *OsMADS34* may influence grain yield by suppressing the expressions of related genes, and high expressions of *BG1*, *GW8*, *GW2*, and *GL7* in the *m34-z* mutant may be important factors for smaller grain from the *m34-z* spb (**Figure 10**; **Supplementary Figure 4**). In addition, eating and cooking quality (ECQ) is mainly affected by the AC, GC, and GT (Tian et al., 2009), and these qualities were altered in the grains from the spb in the *m34-z* mutant, indicating that the mutation of *OsMADS34* affected the ECQ. These phenotypic differences between the *m34-z* and earlier reported mutants may be due to the different genetic backgrounds and the different mutated sites in the target gene. The nucleotides in *osmads34-1* were changed from TCA to AAG, causing a frame-shift mutation (Gao et al., 2010). No obvious expression change of *OsMADS34* was observed in *osmads34-1* compared with the wild-type. *pap2-1* and *los* were from an insertion of Tos17 and T-DNA in the *OsMADS34*, respectively (Kobayashi et al., 2010; Lin et al., 2014). While *m34-z* was a natural mutant allele of *OsMADS34*, had a 1014-bp deletion, resulting in a premature translation stop, and much lower expressions were detected in *m34-z* compared with the wild-type. Meantime, the *m34-z* mutant was derived from the japonica variety ZH11, but the *osmads34-1* and *pap2-1* mutants were isolated from *japonica* variety 9552 and Nipponbare, respectively. These results may be responsible for different phenotypic defects that were not found in the reported *osmads34* mutants. Taken together, these findings showed that *OsMADS34* not only influences grain size, grain yield, and panicle architecture, but also affects ECQ in rice. This work provides new insight into the functions of *OsMADS34* and MADS-box family genes, which are involved in the regulation of floral development in rice.

### *OsMADS34* Determines Glume Fate in Rice

In grass, glumes (rudimentary glume and sterile lemma) are unique structures and account for some of the dramatic morphological variations in spikelets (Zanis, 2007; Li et al., 2009). In the *m34-z* mutant, most of the spikelets developed larger rudimentary glumes and sterile lemmas, compared with wild-type spikelets. The rudimentary glume and sterile lemma in the *m34-z* mutant exhibited similar protrusions and trichomes, and had a similar number of vascular bundles as wild-type. However, no abnormities were observed in the rudimentary glumes of reported *osmads34* mutants. The expression of *OsMADS1*, *OsMADS14*, *OsMADS15*, and *DL* was completely absent in both the rudimentary glumes and sterile lemmas of the wild-type, but these genes were expressed in the rudimentary glumes and sterile lemmas of the *m34-z* mutant. Transcripts of *OsMADS6* were found only in the paleae in the wild-type and the *m34-z* mutant. These results indicated that both rudimentary glumes and sterile lemmas acquired the lemma identity. Meantime, *G1* and *ASP1* were maily expressed in rudimentary glumes and/or sterile lemmas, and restrained the elongation of glumes (Yoshida et al., 2009, 2012). *G1* and *ASP1* transcripts were increased in the *m34-z* mutant, which were consistent of enlarged glumes. Together, with the abundant expression of *OsMADS34* in the glumes, our results revealed that *OsMADS34* not only determined the sterile lemma fate, but also controlled the identity of rudimentary glume.

A putative ancestor of *Oryza* species may have had a spikelet that contained a terminal floret and two lateral florets (Arber, 1934; Takeoka et al., 1993; Kellogg, 2009; Ren et al., 2013). The two lateral florets may have degenerated into the lemmas and the sterile lemma may be morphological modification of the lemmas during evolution (Arber, 1934; Takeoka et al., 1993; Kellogg, 2009; Ren et al., 2013; Lin et al., 2014). In the *m34-z*, *g1*/*ele*, *eg1*, and *asp1* mutants, the sterile lemmas are elongated and transformed into lemma-like organs, acquiring the lemma identity, which supports this hypothesis. Interestingly, the rudimentary glumes were enlarged and their identity was changed in the *m34-z* and *asp1* mutants. The epidermal structure of the rudimentary glumes in the *asp1* mutant was similar to that of the wild-type sterile lemma, whereas the epidermal structure of the rudimentary glumes in the *m34-z* mutant resembled that of the wild-type lemma. In addition, the sterile lemma in the *mfs1* mutant was transformed into a rudimentary glume-like organ and acquired rudimentary glume identity, which indicates that the rudimentary glume and sterile lemma may be homologous organs and that the rudimentary glume and sterile lemma may be severely reduced bract structures (Schmidt and Ambrose, 1998; Terrell et al., 2001; Hong et al., 2010; Ren et al., 2013). In maize and wheat, the bract-like glume organs are similar to the lemma in size and structure (Kellogg, 2001; Yoshida et al., 2009; Ren et al., 2013), whereas it is severely reduced and equivalent to the rudimentary glume in *Oryza* (Takeoka et al., 1993; Bommert et al., 2005; Li et al., 2009; Yoshida et al., 2009; Hong et al., 2010; Ren et al., 2013). Therefore, these results further suggest that the rudimentary glume, sterile lemma, and the lemma may be homologous organs.

### OsMADS34 Is a Grass-Specific E-Function Gene

In grass, functional analysis and morphological evolutionary studies have shown that *OsMADS34* plays an important role in the spikelet development and has distinct functions from the E-class genes in Arabidopsis (Gao et al., 2010; Zhang and Yuan, 2014). The transcripts of E-class genes are mainly detected in floral organs in Arabidopsis, but *OsMADS34* also belongs to the E-class MADS-box family and is expressed in all vegetative and reproductive organs (Rounsley et al., 1995; Pelaz et al., 2000; Gao et al., 2010) (**Figure 9A**). In addition, loss of function of *OsMADS34* leads to various defects, including dwarf phenotypes and abnormal inflorescence architecture and spikelet morphology (Gao et al., 2010; Kobayashi et al., 2010) (**Figures 1** and **2**; **Supplementary Figure 2B–C**), which are different from the defects that occur due to loss of function of other E-class genes, including *OsMADS1*, *OsMADS5*, *OsMADS7*, and *OsMADS8* (Pelucchi et al., 2002; Cui et al., 2010). The *osmads34 osmads1* double mutant shows more defects of the inner floral organs than those of the single mutants, indicating that *OsMADS34* and *OsMADS1* redundantly regulate the identity of the inner three whorls of floral organs, but independently control spikelet and inflorescence morphology in rice (Gao et al., 2010). In this study, the *m34-z* mutant exhibited uniquely larger rudimentary glumes and the rudimentary glume had acquired the lemma identity (**Figures 1** and **2**), which was different from the reported phenotype of the *osmads34* mutants. Interestingly, our results also showed that *OsMADS34* affected grain size by promoting cell expansion in the hulls. *OsMADS34* negatively regulated the expression of genes that are closely associated with grain yield (**Supplementary Figure 4**), suggesting that *OsMADS34* may affect grain yield by suppressing the expression of related genes. These findings reveal that *OsMADS34* exhibits unique functions that are distinct from the reported functions of *OsMADS34* and other SEP homologs in rice and Arabidopsis. In grasses, further research into the functions of *OsMADS34* and its orthologs will facilitate a better understanding of the genetic and evolutionary mechanisms and regulatory networks of spikelet development, which may provide a new opportunity to improve grain yield and quality in rice breeding.

## Author Contributions

Experimental design: QQ and LG; Experiments: DR, YR, YL, ZL, QX, LW, ZQ, JH, and GC; Data analysis: DX, DZ, GZ, LZ, ZG, and GD; Manuscript preparation: DR and QQ.

## Conflict of Interest Statement

The authors declare that the research was conducted in the absence of any commercial or financial relationships that could be construed as a potential conflict of interest.

The reviewer JG and handling Editor declared their shared affiliation, and the handling Editor states that the process nevertheless met the standards of a fair and objective review.

## Footnotes

1 http://rgp.dna.affrc.go.jp/

2 http://www.gramene.org

## References

[B1] Ambrose B. Lerner D. Ciceri P. Padilla C. Yanofsky M. Schmidt R. (2000). Molecular and genetic analyses of the silky1 gene reveal conservation in floral organ specification between eudicots and monocots. *Mol. Cell* 5 569–579. 10.1016/S1097-2765(00)80450-5 10882141

[B2] Arber A. (1934). *The Gramineae: A Study of Cereal, Bamboo, and Grasses*. Cambridge: Cambridge University Press.

[B3] Ashikari M. Sakakibara H. Lin S. Yamamoto T. Takashi T. Nishimura A. (2005). Cytokinin oxidase regulates rice grain production. *Science* 309 741–745. 10.1126/science.1113373 15976269

[B4] Bommert P. Satoh-Nagasawa N. Jackson D. Hirano H. Y. (2005). Genetics and evolution of inflorescence and flower development in grasses. *Plant Cell Physiol.* 46 69–78. 10.1093/pcp/pci504 15659432

[B5] Chen Y. Xu Y. Luo W. Li W. Chen N. Zhang D. (2013). The F-box protein osFBK12 targets OsSAMS1 for degradation and affects pleiotropic phenotypes, including leaf senescence, in rice. *Plant Physiol.* 163 1673–1685. 10.1104/pp.113.224527 24144792 PMC3850201

[B6] Coen E. Meyerowitz E. (1991). The war of the whorls: genetic interactions controlling flower development. *Nature* 353 31–37. 10.1038/353031a0 1715520

[B7] Cui R. Han J. Zhao S. Su K. Wu F. Du X. (2010). Functional conservation and diversification of class E floral homeotic genes in rice (*Oryza sativa*). *Plant J.* 6 767–781. 10.1111/j.1365-313X.2009.04101.x 20003164

[B8] Ditta G. Pinyopich A. Robles P. Pelaz S. Yanofsky M. (2004). The SEP4 gene of *Arabidopsis thaliana* functions in floral organ and meristem identity. *Curr. Biol.* 14 1935–1940. 10.1016/j.cub.2004.10.028 15530395

[B9] Dreni L. Jacchia S. Fornara F. Fornari M. Ouwerkerk P. An G. (2007). The D-lineage MADS-box gene OsMADS13 controls ovule identity in rice. *Plant J.* 52 690–699. 10.1111/j.1365-313X.2007.03272.x 17877710

[B10] Ferrario S. Immink R. Shchennikova A. Busscher-Lange J. Angenent G. (2003). The MADS box gene FBP2 is required for SEPALLATA function in petunia. *Plant Cell* 15 914–925. 10.1105/tpc.010280 12671087 PMC152338

[B11] Fujita D. Trijatmiko K. Taglea A. Sapasap M. Koide Y. Sasaki K. (2013). NAL1 allele from a rice landrace greatly increases yield in modern indica cultivars. *Proc. Natl. Aacd. Sci. U.S.A.* 10 20431–20436. 10.1073/pnas.1310790110 PMC3870739 24297875

[B12] Gao X. Liang W. Yin C. Ji S. Wang H. Su X. (2010). The SEPALLATA-like gene OsMADS34 is required for rice inflorescence and spikelet development. *Plant Physiol.* 153 728–740. 10.1104/pp.110.156711 20395452 PMC2879775

[B13] Guo L. Gao Z. Qian Q. (2014). Application of resequencing to rice genomics, functional genomics and evolutionary analysis. *Rice* 7:4 10.1186/s12284-014-0004-7 PMC4086445 25006357

[B14] Hong L. Qian Q. Zhu K. Tang D. Huang Z. Gao L. (2010). ELE restrains empty glumes from developing into lemmas. *J. Genet. Genomics* 37 101–115. 10.1016/S1673-8527(09)60029-1 20227044

[B15] Hu J. Wang Y. Fang Y. Zeng L. Xu J. Yu H. (2015). A rare allele of GS2 enhances grain size and grain yield in rice. *Mol Plant* 8 1455–1465. 10.1016/j.molp.2015.07.002 26187814

[B16] Hu Y. Liang W. Yin C. Yang X. Ping B. Li A. (2015). Interactions of OsMADS1 with floral homeotic genes in rice flower development. *Mol. Plant* 8 1366–1384. 10.1016/j.molp.2015.04.009 25917758

[B17] Huang X. Qian Q. Liu Z. Sun H. He S. Luo D. (2009). Natural variation at the DEP1 locus enhances grain yield in rice. *Nat. Genet.* 41 494–497. 10.1038/ng.352 19305410

[B18] Ikeda K. Ito M. Nagasawa N. Kyozuka J. Nagato Y. (2007). Rice ABERRANT PANICLE ORGANIZATION 1, encoding an F-box protein, regulates meristem fate. *Plant J.* 51 1030–1040. 10.1111/j.1365-313X.2007.03200.x 17666027

[B19] Jeon J. Jang S. Lee S. Nam J. Kim C. Lee S. (2000). leafy hull sterile1 Is a homeotic mutation in a rice MADS box gene affecting rice flower development. *Plant Cell* 12 871–884. 10.1105/tpc.12.6.871 10852934 PMC149090

[B20] Jiang Y. Bao L. Jeong S. Kim S. Xu C. Li X. (2012). XIAO is involved in the control of organ size by contributing to the regulation of signaling and homeostasis of brassinosteroids and cell cycling in rice. *Plant J.* 70 398–408. 10.1111/j.1365-313X.2011.04877.x 22151303

[B21] Kater M. Dreni L. Colombo L. (2006). Functional conservation of MADS-box factors controlling floral organ identity in rice and *Arabidopsis*. *J. Exp. Bot.* 57 3433–3444. 10.1093/jxb/erl097 16968881

[B22] Kellogg E. (2001). Evolutionary history of the grasses. *Plant Physiol.* 125 1198–1205. 10.1104/pp.125.3.1198 11244101 PMC1539375

[B23] Kellogg E. (2009). The evolutionary history of Ehrhartoideae, Oryzeae, and *Oryza*. *Rice* 2 1–14. 10.1007/s12284-009-9022-2

[B24] Kobayashi K. Maekawa M. Miyao A. Hirochika H. Kyozuka J. (2010). PANICLE PHYTOMER2 (PAP2), encoding a SEPALLATA subfamily MADS-box protein, positively controls spikelet meristem identity in rice. *Plant Cell Physiol.* 51 47–57. 10.1093/pcp/pcp166 19933267 PMC2807174

[B25] Kobayashi K. Yasuno N. Sato Y. Yoda M. Yamazaki R. Kimizu M. (2012). Inflorescence meristem identity in rice is specified by overlapping functions of three AP1/FUL-like MADS box genes and PAP2, a SEPALLATA MADS box gene. *Plant Cell* 24 1848–1859. 10.1105/tpc.112.097105 22570445 PMC3442573

[B26] Komatsu M. Chujo A. Nagato Y. Shimamoto K. Kyozuka J. (2003). FRIZZY PANICLE is required to prevent the formation of axillary meristems and to establish floral meristem identity in rice spikelets. *Development* 130 3841–3850. 10.1242/dev.00564 12835399

[B27] Lee D. An G. (2012). Two AP2 family genes, Supernumerary bract (SNB) and Osindeterminate spikelet 1 (OsIDS1), synergistically control inflorescence architecture and floral meristem establishment in rice. *Plant J.* 69 445–461. 10.1111/j.1365-313X.2011.04804.x 22003982

[B28] Lee D. Lee J. Moon S. Park S. An G. (2007). The rice heterochronic gene SUPERNUMERARY BRACT regulates the transition from spikelet meristem to floral meristem. *Plant J.* 49 64–78. 10.1111/j.1365-313X.2006.02941.x 17144896

[B29] Li F. Liu W. Tang J. Chen J. Tong H. Hu B. (2010). Rice DENSE AND ERECT PANICLE 2 is essential for determining panicle outgrowth and elongation. *Cell Res.* 20 838–849. 10.1038/cr.2010.69 20502443

[B30] Li H. Liang W. Hua Y. Zhu L. Yin C. Xu J. (2011). Rice MADS6 interacts with the floral homeotic genes SUPERWOMAN1, MADS3, MADS58, MADS3, and DROOPING LEAF in specifying floral organ identities and meristem fate. *Plant Cell* 23 2536–2552. 10.1105/tpc.111.087262 21784949 PMC3226212

[B31] Li H. Liang W. Jia R. Yin C. Zong J. Kong H. (2010). The AGL6-like gene OsMADS6 regulates floral organ and meristem identities in rice. *Cell Res.* 20 299–313. 10.1038/cr.2009.143 20038961

[B32] Li H. Xue D. Gao Z. Yan M. Xu W. Xin Z. (2009). A putative lipase gene EXTRA GLUME1 regulates both empty-glume fate and spikelet development in rice. *Plant J.* 57 593–605. 10.1111/j.1365-313X.2008.03710.x 18980657 PMC2667685

[B33] Li Y. Fan C. Xing Y. Jiang Y. Luo L. Sun L. (2011). Natural variation in GS5 plays an important role in regulating grain size and yield in rice. *Nat. Genet.* 243 1266–1270. 10.1038/ng.977 22019783

[B34] Lin X. Wu F. Du X. Shi X. Liu Y. Liu S. (2014). The pleiotropic SEPALLATA-like gene OsMADS34 reveals that the ‘empty glumes’ of rice (*Oryza sativa*) spikelets are in fact rudimentary lemmas. *New Phytol.* 202 689–702. 10.1111/nph.12657 24372518

[B35] Liu C. Xi W. Shen L. Tan C. Yu H. (2009). Regulation of floral patterning by flowering time genes. *Dev. Cell* 16 711–722. 10.1016/j.devcel.2009.03.011 19460347

[B36] Liu L. Tong H. Xiao Y. Che R. Xu F. Hu B. (2015). Activation of Big Grain 1 significantly improves grain size by regulating auxin transport in rice. *Proc. Natl. Aacd. Sci. U.S.A.* 112 11102–11107. 10.1073/pnas.1512748112 PMC4568269 26283354

[B37] Liu S. Hua L. Dong S. Chen H. Zhu X. Jiang J. (2015). OsMAPK6, a mitogen-activated protein kinase, influences rice grain size and biomass production. *Plant J.* 84 672–681. 10.1111/tpj.13025 26366992

[B38] Malcomber S. Kellogg E. (2004). Heterogeneous expression patterns and separate roles of the SEPALLATA gene LEAFY HULL STERILE1 in grasses. *Plant Cell* 16 1692–1706. 10.1105/tpc.021576 15208396 PMC514154

[B39] Malcomber S. Kellogg E. (2005). SEPALLATA gene diversification: brave new whorls. *Trends Plant Sci.* 10 427–435. 10.1016/j.tplants.2005.07.008 16099195

[B40] Mao H. Sun S. Yao J. Wang C. Yu S. Xu C. (2010). Linking differential domain functions of the GS3 protein to natural variation of grain size in rice. *Proc. Natl. Aacd. Sci. U.S.A.* 107 19579–19584. 10.1073/pnas.1014419107 PMC2984220 20974950

[B41] Nagasawa N. Miyoshi M. Sano Y. Satoh H. Hirano H. Sakai H. (2003). SUPERWOMAN1 and DROOPING LEAF genes control floral organ identity in rice. *Development* 130 705–718. 10.1242/dev.00294 12506001

[B42] Ohmori S. Kimizu M. Sugita M. Miyao A. Hirochika H. Uchida E. (2009). MOSAIC FLORAL ORGANS1, an AGL6-like MADS box gene, regulates floral organ identity and meristem fate in rice. *Plant Cell* 21 3008–3025. 10.1105/tpc.109.068742 19820190 PMC2782282

[B43] Pelaz S. Ditta G. Baumann E. Wisman E. Yanofsky M. (2000). B and C floral organ identity functions require SEPALLATA MADS-box genes. *Nature* 405 200–203. 10.1038/35012103 10821278

[B44] Pelucchi N. Fornara F. Favalli C. Masiero S. Lago C. Pè M. (2002). Comparative analysis of rice MADS-box genes expressed during flower development. *Sex. Plant Reprod.* 15 113–122. 10.1007/s00497-002-0151-7

[B45] Qiao Y. Piao R. Shi J. Lee S. Jiang W. Kim B. (2011). Fine mapping and candidate gene analysis of dense and erect panicle 3 , DEP3, which confers high grain yield in rice (*Oryza sativa* L.). *Theor. Appl. Genet.* 122 1439–1449. 10.1007/s00122-011-1543-6 21318372

[B46] Rao Y. Yang Y. Xu J. Li X. Leng Y. Dai L. (2015). EARLY SENESCENCE1 encodes a SCAR-LIKE PROTEIN2 that affects water loss in rice. *Plant Physiol.* 169 1225–1239. 10.1104/pp.15.00991 26243619 PMC4587469

[B47] Ren D. Li Y. Wang Z. Xu F. Guo S. Zhao F. (2012). Identification and gene mapping of a multi-floret spikelet 1 (mfs1) mutant associated with spikelet development in rice. *J. Integr. Agric.* 11 1574–1579. 10.1016/S2095-3119(12)60160-9

[B48] Ren D. Li Y. Zhao F. Sang X. Shi J. Wang N. (2013). MULTI-FLORET SPIKELET1, which encodes an AP2/ERF protein, determines spikelet meristem fate and sterile lemma identity in rice. *Plant Physiol.* 162 872–884. 10.1104/pp.113.216044 23629832 PMC3668076

[B49] Ren D. Rao Y. Huang L. Leng Y. Hu J. Lu M. (2016). Fine mapping identifies a new QTL for brown rice rate in rice (*Oryza Sativa* L.). *Rice* 9 4 10.1186/s12284-016-0076-7 PMC4742455 26847792

[B50] Ren D. Rao Y. Wu L. Xun Q. Li Z. Yu H. (2015). The pleiotropic ABNORMAL FLOWER AND DWARF1 affects plant height, floral development and grain yield in rice. *J. Integr. Plant Biol.* 58 529–539. 10.1111/jipb.12441 PMC5064741 26486996

[B51] Rounsley S. Ditta G. Yanofsky M. (1995). Diverse roles for MADS box genes in *Arabidopsis* development. *Plant Cell* 7 1259–1269. 10.1105/tpc.7.8.1259 7549482 PMC160949

[B52] Schmidt R. Ambrose B. (1998). The blooming of grass flower development. *Curr. Opin. Plant Biol.* 1 60–67. 10.1016/S1369-5266(98)80129-5 10066562

[B53] Song X. Huang W. Shi M. Zhu M. Lin H. (2007). A QTL for rice grain width and weight encodes a previously unknown RING-type E3 ubiquitin ligase. *Nat. Genet.* 39 623–630. 10.1038/ng2014 17417637

[B54] Su Y. Rao Y. Hu S. Yang Y. Gao Z. Zhang G. (2011). Map-based cloning proves qGC-6, a major QTL for gel consistency of japonica/indica cross, responds by Waxyin rice (*Oryza sativa* L.). *Theor. Appl. Genet.* 123 859–867. 10.1007/s00122-011-1632-6 21698394

[B55] Takeoka Y. Shimizu M. Wada T. (1993). “Panicles,” in *Science of the Rice Plant*, Vol. I, eds Matsuo T. Hoshikawa K. (Tokyo: Nobunkyo), 295–326.

[B56] Terrell E. Peterson P. Wergin W. (2001). Epidermal features and spikelet micromorphology in *Oryza* and related genera (Poaceae: Oryzeae). *Smithsonian Contr. Bot.* 91 1–50. 10.5479/si.0081024X.91

[B57] Theissen G. Saedler H. (2001). Plant biology. Floral quartets. *Nature* 409 469–471. 10.1038/35054172 11206529

[B58] Tian Z. Qian Q. Liu Q. Yan M. Liu X. Yan C. (2009). Allelic diversities in rice starch biosynthesis lead to a diverse array of rice eating and cooking qualities. *Proc. Natl. Aacd. Sci. U.S.A.* 106 21760–21765. 10.1073/pnas.0912396106 PMC2793318 20018713

[B59] Wang E. Wang J. Zhu X. Hao W. Wang L. Li Q. (2008). Control of rice grain-filling and yield by a gene with a potential signature of domestication. *Nat. Genet.* 40 1370–1374. 10.1038/ng.220 18820698

[B60] Wang K. Tang D. Hong L. Xu W. Huang J. Li M. (2010). DEP and AFO regulate reproductive habit in rice. *PLoS Genet.* 6:e1000818 10.1371/journal.pgen.1000818 PMC2809758 20107517

[B61] Wang S. Li S. Liu Q. Wu K. Zhang J. Wang S. (2015). The OsSPL16-GW7 regulatory module determines grain shape and simultaneously improves rice yield and grain quality. *Nat. Genet.* 47 949–954. 10.1038/ng.3352 26147620

[B62] Wang S. Wu K. Yuan Q. Liu X. Liu Z. Lin X. (2012). Control of grain size,shape and quality by OsSPL16 in rice. *Nat. Genet.* 44 950–955. 10.1038/ng.2327 22729225

[B63] Wang Y. Xiong G. Hu J. Jiang L. Yu H. Xu J. (2015). Copy number variation at the GL7 locus contributes to grain size diversity in rice. *Nat. Genet.* 47 944–948. 10.1038/ng.3346 26147619

[B64] Wu X. Tang D. Li M. Wang K. Cheng Z. (2013). Loose Plant Architecture1, an INDETERMINATE DOMAIN protein involved in shoot Gravitropism, regulates plant architecture in rice. *Plant Physiol.* 161 317–329. 10.1104/pp.112.208496 23124325 PMC3532263

[B65] Yamaguchi T. Hirano H. (2006). Function and diversification of MADS-box genes in rice. *Scientific World J.* 6 1923–1932. 10.1100/tsw.2006.320 PMC5917342 17205197

[B66] Yamaguchi T. Lee D. Miyao A. Hirochika H. An G. Hirano H. (2006). Functional diversification of the two C-class MADS box genes OSMADS3 and OSMADS58 in *Oryza sativa*. *Plant Cell* 18 15–28. 10.1105/tpc.105.037200 16326928 PMC1323481

[B67] Yoshida A. Ohmori Y. Kitano H. Taguchi-Shiobara F. Hirano H. (2012). ABERRANT SPIKELET AND PANICLE1, encoding a TOPLESS-related transcriptional co-repressor, is involved in the regulation of meristem fate in rice. *Plant J.* 70 327–339. 10.1111/j.1365-313X.2011.04872.x 22136599

[B68] Yoshida A. Suzaki T. Tanaka W. Hirano H. (2009). The homeotic gene long sterile lemma (G1) specifies sterile lemma identity in the rice spikelet. *Proc. Natl. Aacd. Sci. U.S.A.* 106 20103–20108. 10.1073/pnas.0907896106 PMC2775035 19901325

[B69] Yun D. Liang W. Dreni L. Yin C. Zhou Z. Kater M. (2013). OsMADS16 genetically interacts with OsMADS3 and OsMADS58 in specifying floral patterning in rice. *Mol. Plant* 6 743–756. 10.1093/mp/sst003 23300256

[B70] Zanis M. (2007). Grass spikelet genetics and duplicate gene comparisons. *Int. J. Plant Sci.* 168 93–110. 10.1086/509787

[B71] Zhang D. Yuan Z. (2014). Molecular control of grass inflorescence development. *Annu. Rev. Plant Biol.* 65 14.1–14.26 10.1146/annurev-arplant-050213-040104 24471834

[B72] Zuo J. Li J. (2013). Molecular dissection of complex agronomic traits of rice: a team effort by Chinese scientists in recent years. *Natl. Sci. Rev.* 1 253–276. 10.1093/nsr/nwt004

