# Supplementary material for: Regulatory Role of OsMADS34 in the Determination of Glumes Fate, Grain Yield, and Quality in Rice
Source: Front Plant Sci. 2016 Dec 15;7:1853. doi: 10.3389/fpls.2016.01853 (PMC5156729; doi:10.3389/fpls.2016.01853)
Supplement: Supplementary file 1 [file Data_Sheet_1.pdf]

SUPPLEMENTAL FIGURES

Supplemental Figure 1

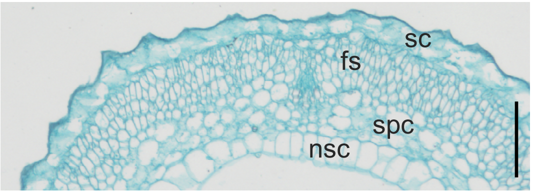

Supplemental Figure 1. Paraffin section analysis of the lemma in the wild type at heading stage. nsc, nonsilicified cells; spc, spongy parenchymatous cells; fs, fibrous sclerenchyma; sc, silicified cells. Bars = 100 μm.

Supplemental Figure 2.

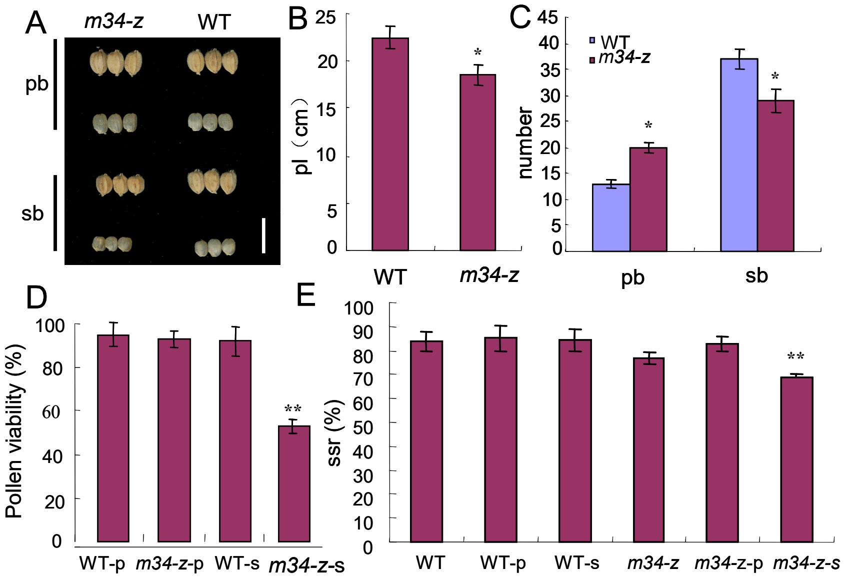

Supplemental Figure 2. The related agronomic characteristics of wild type and *m34-z* mutant. wt, wild type. A, grain sizes in the wild type and *m34-z* mutant. B, panicle length in the wild type and *m34-z* mutant. C, number of primary branches and secondary branches in the wild type and *m34-z* mutant. D, pollen grains in the wild type and *m34-z* mutant. E, seed setting rate in the wild type and *m34-z* mutant; WT indicates the total seed setting rate in E; WT-p indicates the seed setting rate from the primary panicle branches in E; WT-s indicates the seed setting rate from the secondary panicle branches in E; *m34-z* indicates the total seed setting rate in E; *m34-z*-p indicates the seed setting rate from the primary panicle branches in E;

m34-z-s indicates the seed setting rate from the secondary panicle branches in E. WT,wild type; *m34-z*, mutant; pb, primary branches; sb, secondary branches; pl, panicle length; ssr, seed setting rate. Bars = 1cm in A. Error bars indicate SD. **Significant difference at P<0.01 compared with the wild type by Student's test; *Significant difference at P<0.05 compared with the wild type by Student's test.

Supplemental Figure 3

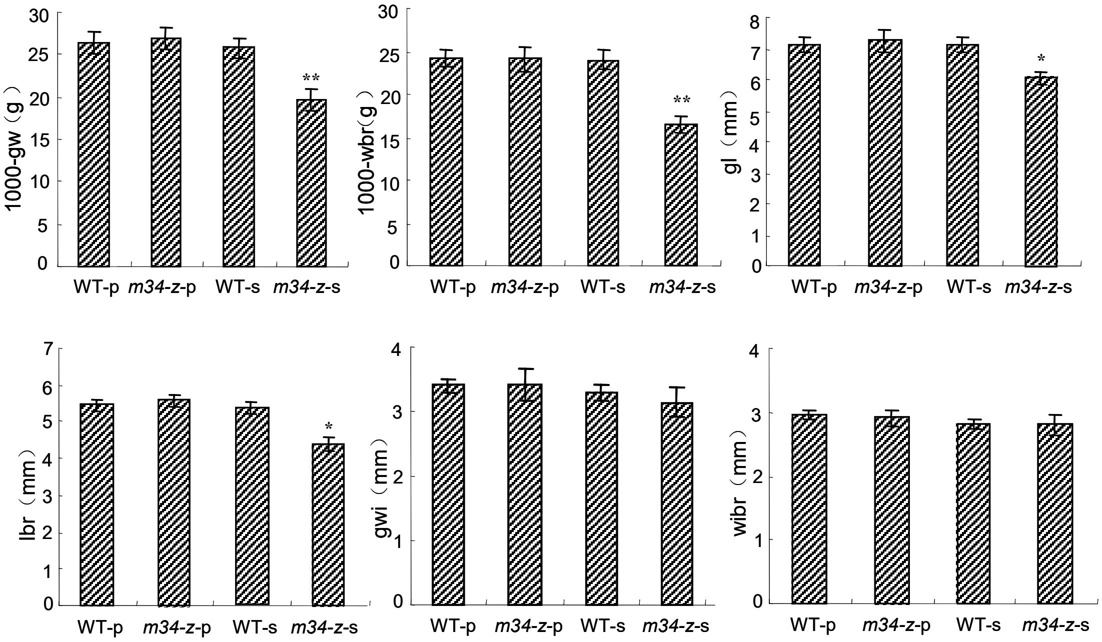

Supplemental Figure 3. size and weight of grain and brown rice in the wild type and *m34-z* mutant. WT-p indicates the seed setting rate from the primary panicle branches; WT-s indicates the seed setting rate from the secondary panicle branches; *m34-z*-p indicates the seed setting rate from the primary panicle branches; *m34-z*-s indicates the seed setting rate from the secondary panicle branches. gw, grain weight; wbr, weight of brown rice; gl, grain length; lbr, length of brown rice; gwi, grain width; wibr, width of brown rice. Error bars indicate SD. **Significant difference at P<0.01 compared with the wild type by Student's test; *Significant difference at P<0.05 compared with the wild type by Student's test.

Supplemental Figure 4

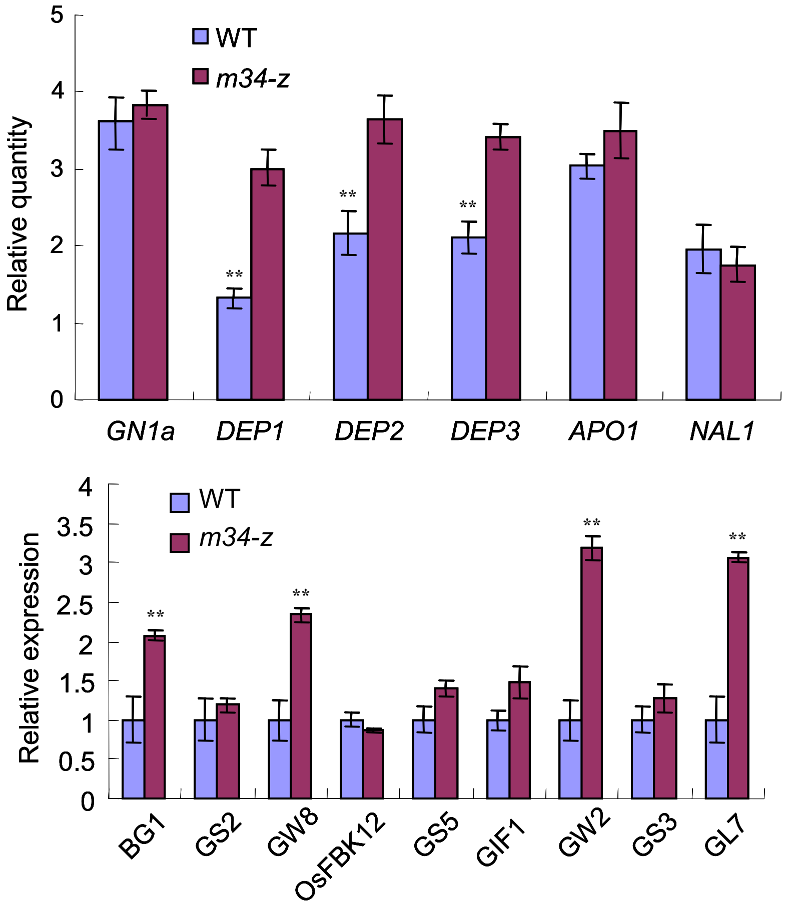

Supplemental Figure 4. Expression of genes related to panicle architecture and grain size. Error bars indicate SD. **Significant difference at $P<0.01$ compared with the wild type by Student's test.

Supplemental Figure 5

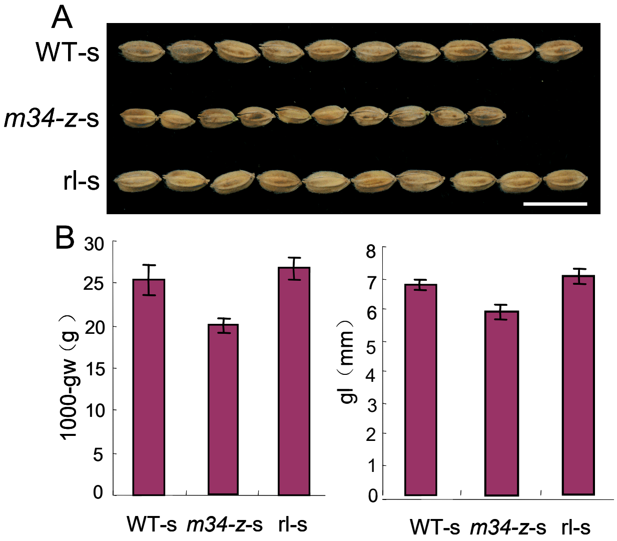

Supplemental Figure 5. complementation test. A, grains of WT-s, *m34-z*-s and rl-s. B, 1000-grain weight and average length of WT-s, *m34-z*-s and rl-s grains.WT-s indicates grains from the secondary panicle branches in the wild type; *m34-z*-s indicates grains from the secondary panicle branches in the *m34-z* mutant; rl-s indicates grains from the secondary panicle branches in the rescued lines. gw, grain weight; gl, grain length. Error bars indicate SD.

Supplemental Table

Supplemental Table 1. Primers used in the study.

Purpose	Primer name	Sequence
Mapping	M7-1F	GGTCAAATCATCACCTGAC
	M7-1R	CAAGGCTTGCAAGGGAA
	M9-1F	GGAGCAAGAAAAGTTCCC
	M9-1R	CAATGTGTGACGCAATAG
	M16-1F	GGCGTCCCTCTCCCTTTCC
	M16-1R	GCAGCCCTAGCAATTCAGTC
	M17-1F	CCGACACTTCAGGGAATAA
	M17-1R	GCCAGGACTAAACTAAACAG
	M24-1F	GAAGAACCTGCGTATCAAGAC
	M24-1R	CCACACGGAAGCAGAATTAG
	M29-1F	GAGTGGGTGAGAAGGATCAGC
	M29-1R	CAGCAGATTTGTTGGCATCTG
	S8-1F	CTCGACGAGTTGGCTCCAGC
	S8-1R	GAGCATCTCCAACAGCCTCTC
	S21-1F	GTGCCACAACGAGGAATAGG
	S21-1R	GTACAGGACCTATGGCAAGAGC
qRT-PCR	*OsMADS1*-1F	GCTGCAACTACAACTCACAGG
	OsMADS1-1R	TGATGGTGAGCATGAGGGTG
	OsMADS14-1F	CCATTAACGAGCTTCAACGG
	OsMADS14-1R	TGGTATGGATCTGAAGCCTCC
	OsMADS15-1F	AGTACGCCACTGACTCCAGG
	OsMADS15-1R	TGCTGGCCCCTCACATTC
	OsMADS6-1F	CCAACAATGCACTTTCTGAAAC
	OsMADS6-1R	GGAGGCTTGCTGCATGGC
	DL-1F	CCCATCTGCTTACAACCGCTT
	DL-1R	GTTGGAGGTGGAAACCGTCG
	G1-1F	GGCGTCTACTTGCCATTTCTG
	G1-1R	TCGATCAGCATCAAAGCACAG
	FZP-1F	GCATGGCTAATCACGCACTTT
	FZP-1R	CCAAGCCACTCTTCTTGTTCG
	SNB-1F	ACCACGAAGTAGGGAACGACTGGG
	SNB-1R	CAGCCAATAAGTCCTCAGTGGCCTG
	OsIDS1-1F	GTCGTCGTCAGTCGAGGCG
	OsIDS1-1R	GCGACTCCACATTGAGATCCA
	MFS1-1F	CGGCTCGTGATCTCGACACGTAC
	MFS1-1R	CACAGCCGGACCAGTGCTCTC
	ASP1-1F	GCTATGTGCTTGGAGCATTG
	ASP1-1R	AGTCCAACGGCTCCACCACA
	GN1a-1F	GTCCACGACGGCGAGCTCAA
	GN1a -1R	TCATGCGAGTGGTGACGTGA

	APO1-1F	TCCGGCAGGTTCTACTGCATG
	APO1-1R	ACGCTGAGACGGCTCTTCTCG
	DEP1-1F	CCGTTTCTCGTTCTGGAT
	DEP1-1R	ATCTGTGCCTCCTTCTCT
	DEP2-1F	TGCAGAGCCTCCAATAGTAGTCCA
	DEP2-1R	GGTACATCAAGCCTCTGTAGTGCAA
	DEP3-1F	TGGTGGACCAAGCTGTATCA
	DEP3-1R	GATGACTTTGACATTTCCACCGT
	NAL1-1F	GATGACTTTGACATTTCCACCGT
	NAL1-1R	GAGTGATTCATTGGTAATGATAA
	OsEXPA1-1F	TGCAGAGCCTCCAATAGTAGTCCA
	OsEXPA1-1R	GGTACATCAAGCCTCTGTAGTGCAA
	OsEXPA2-1F	TTTGGCTATTCTGAGGCTGCT
	OsEXPA2-1R	TGGTCCCAAAAGCACAAGAGT
	OsEXPB1a-1F	GCAGTGCAGAGTTGCGGTAAATTG
	OsEXPB1a-1R	ATCGACGACGACACAGTCACATCA
	OsEXPB9-1F	TGCCAGACGCCATCTACGTATCAA
	OsEXPB9-1R	TGGCGCAGATATACAAGCGCAACT
	OsEXPB10-1F	ATTGGAAGGCCAACGCTCTCTACA
	OsEXPB10-1R	TTCTGCTCACTCCACAAACCTAGC
	OsEXPB11-1F	GCAGTGCAGAGTTGCGGTAAATTG
	OsEXPB11-1R	ATCGACGACGACACAGTCACATCA
	MS1-1F	CAACCAGAGCACTTCTTCC
	MS1-1R	TGAAGCTGAAACGGTAGC
	*OsMADS34*q-1F	GCTTAAGACGCTTGAGAGATACC
	*OsMADS34*q-1R	GACTACTTGACTCTCAAGCTGCTC
	ACTIN-F	GACCCAGATCATGTTTGAGACCT
	ACTIN-R	CAGTGTGGCTGACACCATCAC
GUS staining	pro*OsMADS34*-GUS-1F	GGGCCCAACATACTCAAGGC
	pro*OsMADS34*-GUS-1R	CTCGATCTCCCGCTCGAGA
Subcellular localization	*OsMADS34*OE-1F	ATGGGGCGAGGCAAGGTG
	*OsMADS34*OE-1R	GGCCATCCACTCAGGAGGATAAC
Transcription activity analysis	*OsMADS34*OE-2F	ATGGGGCGAGGCAAGGTG
	*OsMADS34*OE-2R	CTAGGCCATCCACTCAGGAGGAT
Complementation test	*OsMADS34*com-1F	ATGGGGCGAGGCAAGGTG
	*OsMADS34*com-1R	CTAGGCCATCCACTCAGGAGGAT

Supplemental Table 2. Comparisons of the physicochemical characteristics of milled rice between the wild type and *m34-z* mutant.

Physicochemical characteristics	WT-p	WT-s	*m34-z*-p	*m34-z*-s
Amylosecontent (%)	17.83±0.23	18.12±0.30	17.96±0.47	14.89±0.19
Gel consistency (mm)	97±1.00	96±1.00	96±1.00	89±0.50
Gelatinization temperature	6	6	6	6

Supplemental Table 2. Comparisons of the physicochemical characteristics of milled rice between the wild type and *m34-z* mutant. WT-p, grains from the primary panicle branches in the wild type; WT-s, grains from the secondary panicle branches in the wild type panicle; *m34-z*-p, grains from the primary panicle branches in the *m34-z* mutant; *m34-z*-s, grains from the secondary panicle branches in the *m34-z* mutant.
